# Differentiation of Retinal Glial Cells From Human Embryonic Stem Cells by Promoting the Notch Signaling Pathway

**DOI:** 10.3389/fncel.2019.00527

**Published:** 2019-12-03

**Authors:** Sook Hyun Chung, Weiyong Shen, Kathryn C. Davidson, Alice Pébay, Raymond C. B. Wong, Belinda Yau, Mark Gillies

**Affiliations:** ^1^Save Sight Institute, Department of Clinical Ophthalmology and Eye Health, The University of Sydney, Sydney, NSW, Australia; ^2^Centre for Eye Research Australia, Royal Victorian Eye and Ear Hospital, East Melbourne, VIC, Australia; ^3^Department of Anatomy and Neuroscience, The University of Melbourne, Parkville, VIC, Australia; ^4^Department of Surgery, The University of Melbourne, Parkville, VIC, Australia; ^5^Shenzhen Eye Hospital, Shenzhen, China

**Keywords:** human pluripotent cells, retinal glial cells, notch signaling pathway, differentiation, Müller cells in retina

## Abstract

Dysfunction of retinal glial cells, particularly Müller cells, has been implicated in several retinal diseases. Despite their important contribution to retinal homeostasis, a specific way to differentiate retinal glial cells from human pluripotent stem cells has not yet been described. Here, we report a method to differentiate retinal glial cells from human embryonic stem cells (hESCs) through promoting the Notch signaling pathway. We first generated retinal progenitor cells (RPCs) from hESCs then promoted the Notch signaling pathway using Notch ligands, including Delta-like ligand 4 and Jagged-1. We validated glial cell differentiation with qRT-PCR, immunocytochemistry, western blots and fluorescence-activated cell sorting as we promoted Notch signaling in RPCs. We found that promoting Notch signaling in RPCs for 2 weeks led to upregulation of glial cell markers, including glial fibrillary acidic protein (GFAP), glutamine synthetase, vimentin and cellular retinaldehyde-binding protein (CRALBP). Of these markers, we found the greatest increase in expression of the pan glial cell marker, GFAP. Conversely, we also found that inhibition of Notch signaling in RPCs led to upregulation of retinal neuronal markers including cone-rod homeobox (CRX) and orthodenticle *homeobox* 2 (OTX2) but with little expression of GFAP. This retinal glial differentiation method will help advance the generation of stem cell disease models to study the pathogenesis of retinal diseases associated with glial dysfunction such as macular telangiectasia type 2. This method may also be useful for the development of future therapeutics such as drug screening and gene editing using patient-derived retinal glial cells.

## Introduction

Retinal glial cells consist of astrocytes, Müller cells and microglia. Their functions include providing anatomical, metabolic and functional support for neurons and surrounding compartments, phagocytosing of cell debris, taking up neurotransmitters and ions and maintaining the blood retinal barrier (Vecino et al., 2016). Activation of retinal glia (gliosis) and dysfunction have been found in animal models of retinal diseases as well as studies on post mortem human tissues (Rungger-Brandle et al., 2000; Fletcher et al., 2005; Powner et al., 2010, 2013; Arroba et al., 2011; Ly et al., 2011; Ma et al., 2012).

Müller cell deficiency is a striking and consistent pathological feature observed in *post mortem* specimens of eyes with macular telangiectasia type 2 (Mactel2), a bilateral macular disease that damages the central vision by causing characteristic alterations in retinal photoreceptors and blood vessels (Charbel Issa et al., 2013; Wu et al., 2013). Previous studies found absence of Müller cell markers, including vimentin, glutamine synthetase (GS) and cellular retinaldehyde binding protein (CRALBP), in the affected macular region of MacTel2 donor eyes (Powner et al., 2010, 2013). Selective disruption of Müller cells in transgenic mice leads to photoreceptor degeneration, retinal vascular leak, and, later, the development of subretinal neovascularization, all of which are important features of MacTel2 in humans (Shen et al., 2012, 2014). These observations indicate that Müller cell dysfunction may play an important role in the pathogenesis of MacTel2.

The rapid progress in stem cell research has made it possible to generate several retinal cell types, including retinal pigment epithelial (RPE) cells, photoreceptors and ganglion cells, from human pluripotent stem cells (Lamba et al., 2010; Gill et al., 2014, 2016; Lidgerwood et al., 2016, 2018). Retinal glial cell differentiation has previously been described in a method for differentiation of retinal organoids (Sasai et al., 2012; Zhong et al., 2014). However, these organoids consist of heterogenous cell types in suspension culture, which limits the downstream analysis assays that can be performed. To date, there is no report of a differentiation method in adherent culture that specifically produces retinal glial cells. Here, we report a method to differentiate retinal glial cells from human embryonic stem cells (hESCs) by promoting the Notch signaling pathway.

The Notch signaling pathway, which is highly conserved in embryogenesis, regulates cell-fate decisions, such as self-renewal and survival, and cellular differentiation in various organs, including the central and peripheral nervous systems (Gaiano and Fishell, 2002; Taylor et al., 2007). It also promotes glial cell differentiation during retinogenesis (Jadhav et al., 2006a, b) and several animal studies have reported its critical role in driving RPCs to differentiate into retinal glial cells in rodents (Furukawa et al., 2000; Bernardos et al., 2005). When the Notch signaling pathway is activated in retinal progenitor cells (RPCs), the downstream effector genes, including Hairy and Enhancer of Split (Hes) 1 and Hes 5, suppress transcription of pro-neural genes and activate glial-specific genes such as GFAP (Vetter and Moore, 2001). Here, we tested the hypothesis that activation of the Notch signaling pathway in human RPCs will generate human retinal glial cells.

## Materials and Methods

### hESCs Culture, Retinal Progenitor Cell Differentiation and the Notch Signaling Pathway Promotion

Undifferentiated hESCs (WA-09 alias H9, WiCell) were maintained in mTeSR1^TM^ media (Stem Cell Technologies, 85850) without feeders and passaged weekly. hESCs were differentiated into RPCs using a published method with minor modifications (Lamba et al., 2006, 2010). Briefly, hESCs were seeded into Matrigel-coated 6 well plates (around 15–20 /well) and cultured in neural induction media (NIM; DMEM/F12 with 10% Knockout Serum Replacement, B27 and N2) containing a cocktail consisting of recombinant human proteins including insulin-like growth factor-1 (IGF-1, 1 ng/ml, PeproTech, cat# 100-11), Dickkopf Wnt signaling pathway inhibitor (DKK-1, 1 ng/ml, PeproTech, cat# 120–30) and the bone morphogenetic protein antagonist Noggin (R&D systems, 3344-NG-050) for 4 days. We increased the concentrations of all recombinant proteins to 10 ng/ml from the 5th day on wards.

We promoted the Notch signaling pathway in the resultant RPCs by adding recombinant human Notch ligands. RPCs were passaged at 3 weeks of differentiation and re-seeded on matrigel-coated 6 well plates (20–25 clumps/well) and cultured in NIM without N2 supplement but including Notch ligands Delta-like ligand 4 (DLL-4, R&D systems, cat# 15106-D4-050) and Jagged-1 (R&D systems, cat# 1277-JD-050). Media were changed every 2–3 days. All recombinant proteins including IGF-1, Noggin, Dkk-1 for RPC generation and Notch ligands including DLL4 and Jagged-1 for Notch stimulation, were freshly added into culture media every time when the media were changed. The Notch ligand treatment lasted for as long as 6 weeks. Cell were collected for analysis 2, 4, and 6 weeks after promoting the Notch signaling pathway as described below.

### qRT-PCR

Total RNA were extracted from cell pallets using a RNeasy mini kit (Qiagen, 74104) according to the manufacturer’s instructions. The quality and quantity of extracted RNA were assessed with a Bioanalyzer (Agilent). An equal amount of RNA were reverse transcribed into cDNA with SuperScript Vilo cDNA synthesis kit (Invitrogen, 11704050). A SYBR GreenER qPCR Supermix (Invitrogen, 11784-200) was used for qRT-PCR reaction. The PCR cycling temperatures were 95°C for 5 min, 95°C for 10 s, 60°C for 15 s, and 72°C for 20 s followed by a melting curve analysis. A total of 40 cycles were conducted for qRT-PCR. Quantitative analyses were performed by Relative Expression Software Tool 2009 (REST2009) and a built-in analysis software in the PCR machine (Bio-Rad CFX96). The primers used in this study are listed in Table 1.

**TABLE 1:** Primers used in this study.

**Primer**	**Forward (5 - >3)**	**Reverse (5 - >3)**
OCT4	GTG GAG GAA GCT GAC AAC AA	ATT CTC CAG GTT GCC TCT CA
NANOG	CAA AGG CAA ACA ACC CAC TT	TCT GCT GGA GGC TGA GGT AT
CHX10	GGC GAC ACA GGA CAA TCT TTA	TTC CGG CAG CTC CGT TTT C
PAX6	AACGATAACATACCAAGCGTGT	GGTCTGCCCGTTCAACATC
HES1	CCTGTCATCCCCGTCTACAC	CACATGGAGTCCGCCGTAA
HES5	CTCAGCCCCAAAGAGAAAAA	GACAGCCATCTCCAGGATGT
GS	AAGAGTTGCCTGAGTGGAATTTC	AGCTTGTTAGGGTCCTTACGG
CRALBP	TGCAGGCATATTGCTTCATCC	GCTTGACCACATTGTAGGTCG
VIMENTIN	TGCCGTTGAAGCTGCTAACTA	CCAGAGGGAGTGAATCCAGATTA
GFAP	CTGCGGCTCGATCAACTCA	TCCAGCGACTCAATCTTCCTC
CRX	TAT TCT GTC AAC GCC TTG GCC CTA	TGC ATT TAG CCC TCC GGT TCT TGA
RECOVERIN	CCAGAGCATCTACGCCAAGTT	CCGTCGAGGTTGGAATCGAAG

### Immunocytochemistry

Cells growing on plastic coverslips placed in 24 well plates were fixed with 4% paraformaldehyde for 20 min on ice and washed with PBS for times times, with 5 min/time. The fixed cells were permeabilized with 0.1% Triton X100 in PBS followed by blocking with 10% normal donkey serum in PBS for 1 h at room temperature. Primary antibodies were applied over night at 4°C, followed by an incubation with corresponding secondary antibodies-conjugated with Alexa Fluor 488 or 594 at room temperature for 2 h on the next day. The primary antibodies we used in the study are GFAP (DAKO, cat# Z3044), PAX6 (DSHB, cat# pax6), OTX2 (R&D Systems, AF1979), and CRX (Santa Cruz, cat# sc-30150). The stained coverslips were mounted for confocal laser scanning microscopy (Zeiss) as described previously (Shen et al., 2012; Chung et al., 2016).

### Western Blots

Western blots were performed as previously described (Chung et al., 2016). Briefly, proteins were extracted from cell pallets with RIPA buffer (Sigma, cat# R0278) containing protease inhibitor (Roche, cat# 04693159001). The concentrations of extracted cellular proteins were measured using a QuantiPro BCA assay kit (Sigma-Aldrich, cat# QPBCA). Equal amounts of protein were loaded into NuPage Bis-Tris gels (Life Technologies, cat# NP3023BOX) and transferred to a polyvinylidene difluoride membrane with iBlot semi-dry transfer system (Invitrogen, cat# IB21001). The membranes were blocked with 5% BSA in TBST and primary antibody was incubated overnight at 4°C. After incubation with secondary antibodies conjugated with horseradish peroxidase, protein bands were visualized using the G:Box BioImaging systems and quantified using the GeneTools image scanning and analysis package. Protein expression was normalized to α/β-tubulin (rabbit polyclonal, 1:2000; Cell Signaling #2148), which serves as a loading control.

### Fluorescence Activated Cell Sorting (FACS) Analysis

Cells were collected with Accutase (Sigma, cat# A6964) to produce single cell suspension. After washing with PBS for 2 times, we labeled the cells with Fixable Viability Dye eFluor 780 (eBioscience, cat# 65-0865) minutes at 4°C for 30 min in order to label dead cells. The cells were washed 2 times with flow staining buffer (eBioscience, cat# 00-42226) and fixed with IC fixation buffer (eBioscience, cat# 822249) at room temperature for 20 min. Cells were then washed 2 times with ×1 permeabilization buffer (eBioscience, cat# 833356), blocked with 2% normal donkey serum at room temperature for 15 min and labeled with GFAP antibody (Rabbit Polyclonal, 1:500, Dako, cat# Z0334) at room temperature for 1 h. After washing cells 2 times with ×1 permeabilization buffer, GFAP-labeled cells were incubated with a secondary antibody-conjugated with Alexa Fluor 488 (1:1000, Invitrogen, cat# A21206) at room temperature for 40 min. Cells were washed 2 times with flow staining buffer and FACS was performed with LXR Fortessa X-20 (BD). Ten thousand cells were counted per sample and a rabbit IgG was used as a control for FACS analysis in this study. Data was analyzed with FlouJo software.

### Statistical Analysis

Statistical analysis was performed by Student *t*-test with *p* values <0.05 deemed significant. In each characterization study, 3–6 biological replicates were used.

## Results

### Differentiation of H9 hESCs Into RPCs

**FIGURE 1:**
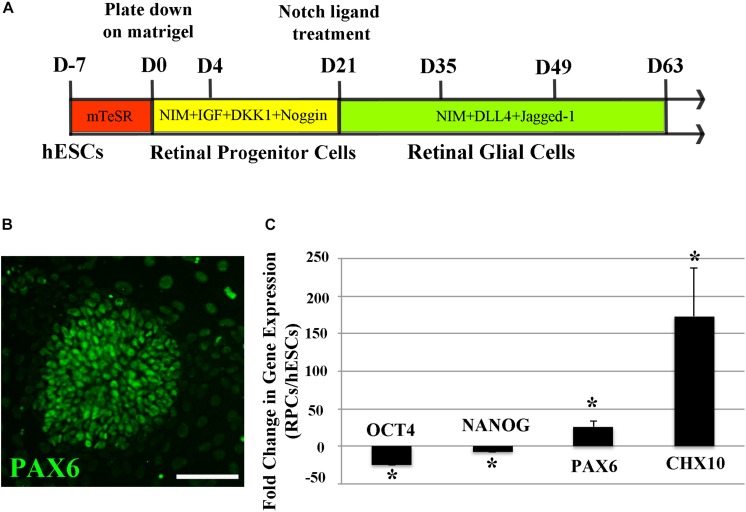
Differentiation of H9 human embryonic stem cells (hESCs) into retinal progenitor cells (RPCs). **(A)** A schematic diagram illustrating the method of differentiating hESCs to RPCs and glial cells. **(B)** Immunocytochemistry results showing formation of PAX6 positive retinal progenitors with rosette-like morphology 15 days after culture in the neural induction medium. **(C)** qRT-PCR analyses 3 weeks after directing hESCs to differentiate into RPCs. Our results indicated significant upregulation of the eye field markers *PAX6* and *CHX10* along with decreased expression of stem cell markers of *OCT4* and *NANOG* after culturing H9 hESCs in the neural induction medium for 3 weeks ^∗^*p* < 0.05 vs. undifferentiated human ESCs, error bars represent SEM, *n* = 3/group. Scale bar: 100 μm.

We first differentiated hESCs into human RPCs using a previously reported method (Lamba et al., 2006, 2010) by culturing them in neural induction media containing recombinant human IGF1, DKK1, and Noggin for 3 weeks (Figure 1A). We performed RT-PCR to study the expression profiles of a panel of stem cell and RPC markers from D0 to D12 of RPC differentiation. We found that pluripotent stem cell markers including *OCT4, NANOG*, and *SOX 2* were suppressed during RPC differentiation, whereas RPC markers including *CHX10, LHX, RX, SIX6*, and *PAX6* were increased as early as 3 days after differentiation (Supplementary Figure S1). hESCs formed rosette-like structures from 10 days after treatment with the cocktail of recombinant human proteins (Figure 1B and Supplementary Figure S2A), consistent with previous reports that the formation of rosette-like structures was one of the characteristic stages of RPC differentiation (Lamba et al., 2006, 2010). By 3 weeks after treatment with Notch ligands, stem cell pluripotency markers including *OCT4* and *NANOG* were markedly suppressed, whereas *PAX6* and *CHX10* were significantly upregulated for more than 50 times than that of undifferentiated ESCs (Figure 1C).

### Long-Term Activation of Notch Signaling in RPCs Leads to Upregulation of Retinal Glial Cell-Related Genes

**FIGURE 2:**
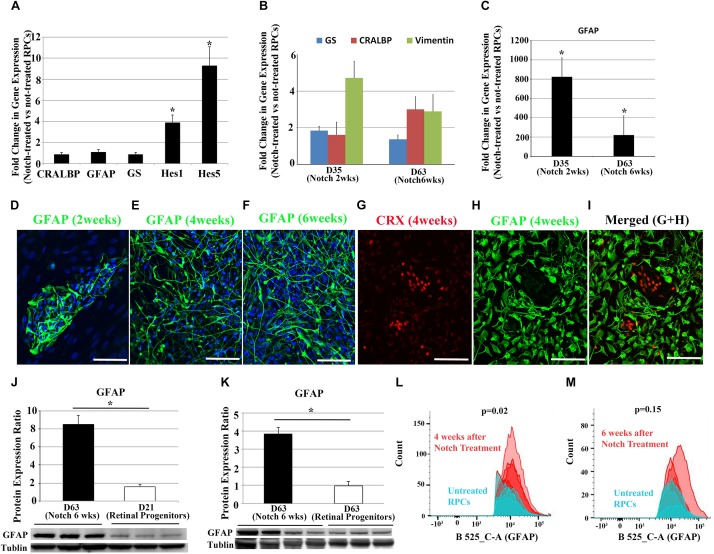
Changes in Notch target genes and glia cell-associated markers after Notch ligand treatment in RPCs. **(A)** qRT-PCR analyses indicate Notch ligand treatment of RPCs for 9 days significantly upregulated the expression of Notch downstream effector genes including *HES1* and *HES5*, but with much less effect on glia cell-associated makers *CRALBP, GFAP* and *GS*. **(B,C)** Gene expression levels of *CRALBP, VIMENTIN, GS* and *GFAP* over the course of Notch ligand treatment. **(D–F)** Immunostaining for the glial cell marker GFAP 2, 4, and 6 weeks after Notch ligand treatment in RPCs. **(G–I)** Double label immunostaining for the photoreceptor precursor marker CRX and glial cell marker GFAP 4 weeks after Notch ligand treatment. Scale bars: 100 μm. **(J,K)** Western blots for GFAP using proteins extracted from un-treated RPCs and those treated with Notch ligands for 6 weeks. *n* = 3–4. **(L,M)** FACS analyses showed increased numbers of GFAP positive cells after treating RPCs with Notch ligands for 4 and 6 weeks. ^∗^*p* < 0.05, error bars represent SEM, *n* ≥ 3.

Once RPC differentiation had been confirmed, we promoted the Notch signaling pathway in RPCs by culturing them with Notch ligands including DLL4 and Jagged-1. qRT-PCR analyses indicated that promoting the Notch signaling pathway with DLL4 and Jagged-1 (both 50 ng/ml) for 9 days significantly increased the expression of Notch downstream genes, *HES1* and *HES5*, while the increases in retinal glial cell-related genes including *CRALBP*, *GFAP*, and *GS* were not significant (Figure 2A), suggesting that the differentiation of human RPCs into retinal glial cells may require longer term Notch signaling activation.

We next treated RPCs for more extended periods ranging from 2 to 6 weeks with a cocktail of DLL4 and Jagged-1 (50 ng/ml). Transcription of glial genes including *GFAP, GS, CRALBP* and *VIMENTIN* started to increase after 2 weeks of Notch promotion when compared with untreated RPCs (Figure 2B). qRT-PCR analysis indicated promoting the Notch signaling pathway significantly increased the expression of *GFAP* from 2 weeks after treatment. By 6 weeks, the level of *GFAP* expression in Notch-treated group was more than 200 times higher than that in un-treated RPCs (Figure 2C). We also noted that there was a decrease in up-regulation of GFAP at 6 weeks compared with 2 weeks after Notch treatment (Figure 2C).

We also studied morphological changes of RPCs after activating the Notch signaling pathway. While bright field images from an Epi-fluorescence microscope revealed relatively consistent morphology throughout the culture (Supplementary Figure S2B), immunohistochemical analysis revealed that GFAP^+^ cells appeared from 2 weeks after treatment and this glia-phenotypic change became more obvious after 4 to 6 weeks of promoting Notch signaling (Figures 2D–F). Interestingly, confocal microscopy showed that most GFAP^+^ cells had long processes 4 and 6 weeks after Notch treatment, which is consistent with the typical morphology of Müller glial cells in the retina (Figures 2E,F). Double labeling with the glial cell marker GFAP and the photoreceptor precursor marker cone-rod homeobox protein (CRX) indicated that most treated RPCs lost this precursor marker of CRX and became positive for the glial cell marker of GFAP 4 weeks after Notch promotion (Figures 2G–I). Consistent with the results of qRT-PCR and immunohistochemistry, Western blots indicated that Notch ligand treatment for 6 weeks significantly increased GFAP expression compared with RPCs cultured in media without Notch ligands (Figures 2J,K). qRT-PCR analyses of Notch receptors and Notch downstream effector genes indicate that the upregulated *HES1* and *HES5* were decreased at 6 weeks (Supplementary Figure S3) compared with 9 days after Notch treatment (Figure 2A). We performed FACS to analyze the number of GFAP^+^ cells 4 and 6 weeks after Notch treatment (Figures 2L,M). Treatment of RPCs with Notch ligands for 4 weeks significantly increased the number of GFAP^+^ cells compared with RPCs cultured in a medium without Notch ligands for the same period (Figure 2L and Supplementary Figure S4). Consistent with our qRT-PCR results revealed in Figure 2C, we also found that the number of GFAP^+^ cells decreased during Notch ligand treatment (Figure 2M and Supplementary Figure S4). Interestingly, 22% of RPCs became GFAP^+^ 4 weeks after culturing in the medium without Notch ligands and the number of GFAP^+^ cells also decreased over time (Figures 2L,M and Supplementary Figure S4), indicating that some un-treated RPCs may undergo spontaneous differentiation without the promotion of the Notch signaling pathway.

### Notch Promotion and Inhibition Have Opposite Effects on Retinal Precursor Cell Differentiation

**FIGURE 3:**
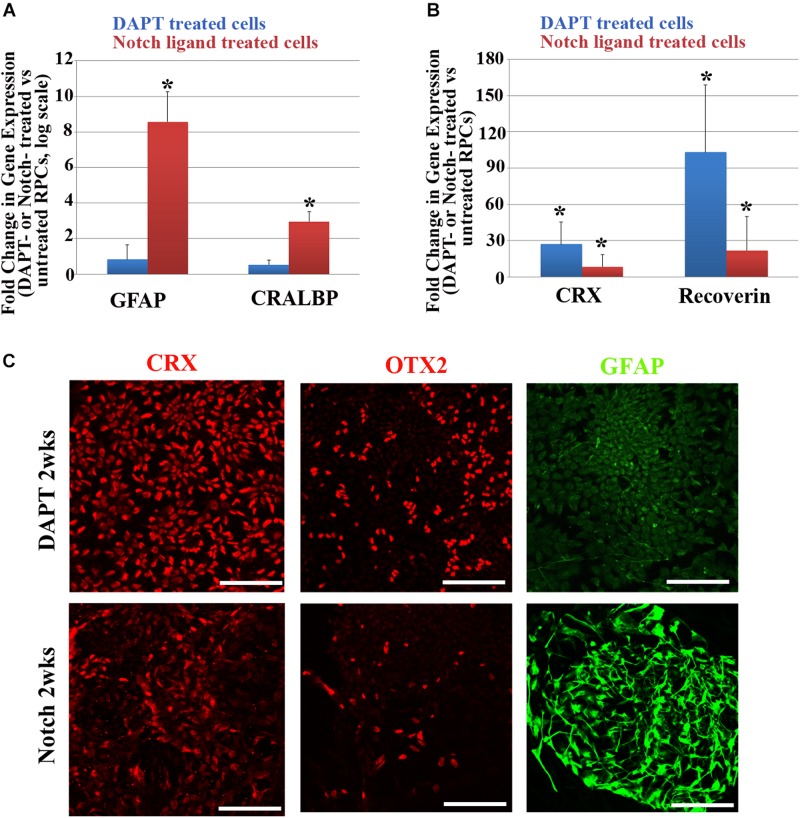
Changes in glial cell- and retinal neuron-associated markers after Notch promotion or inhibition in RPCs. **(A,B)** qRT-PCR analyses of changes in glial cell-associated markers including *GFAP* and *CRALBP* **(A)** and retinal neuron-associated markers including *CRX* and recoverin **(B)** in RPCs 2 weeks after treatment with Notch ligands or Notch inhibitor DAPT. ^∗^*p* < 0.05, RPCs treated with Notch ligands or Notch inhibitor DAPT vs. untreated RPCs, *n* = 3–4/group. **(C)** Immunostaining showing that most RPCs treated with the Notch inhibitor DAPT for 2 weeks were positive for the neuronal markers CRX and OTX2 but they expressed little of the glial cell marker GFAP **(upper panel)**. In contrast, 2 weeks after Notch ligand treatment only a few cells were positive for CRX and OTX2 and the majority of cells became positive for the glial cell marker GFAP **(lower panel)**. Scale bar: 100 μm. Error bars represent SEM.

We also studied the effects of promotion or inhibition of Notch signaling on neuro-gliogenesis further by treating RPCs with Notch ligands (DLL4 and Jagged-1) or a Notch inhibitor DAPT (5 μM in neural induction media) for 2 weeks. Consistent with what we observed earlier, Notch promotion led to upregulation of *GFAP* and *CRALBP* (Figure 3A), whereas Notch inhibition with DAPT in RPCs upregulated the expression of photoreceptor-associated genes, including *CRX* and *RECOVERIN* (Figure 3B). Immunocytochemical studies confirmed that the majority of DAPT-treated RPCs were positive for neuronal markers CRX and OTX2 but mostly negative for GFAP, whereas most Notch ligand-treated RPCs became positive for GFAP with only a few cells positive for CRX and OTX2 (Figure 3C).

## Discussion

In this study we developed a method to differentiate hESCs into human retinal glial cells. Our two-step differentiation method consists of directing hESCs to differentiate first into RPCs, followed by promotion of Notch signaling using DLL4 and Jagged-1 to facilitate retinal glial cell differentiation. We performed qRT-PCR, immnunocytochemistry, Western blots and FACS analyses to study changes in both gene and protein expression of retinal glial cell markers over the course of retinal glial cell differentiation. We found that promoting Notch signaling in RPCs led to upregulation of GFAP and cells displayed Müller-glia like morphology. Our findings suggest that promotion of the Notch signaling pathway in human RPCs leads to differentiation of retinal glial cells.

Müller cells, which account for 90% of macroglial cells in the retina, are the only retinal glial cell type that originates from RPCs (Arroba et al., 2011). Previous cell lineage tracing studies indicate that retinal astrocytes are derived from glial progenitor cells and migrate into the retina through the optic nerve (Stone and Dreher, 1987; Liu et al., 2002, 2004). Microglia cells originate from circulating monocytes as a part of immune defense mechanism (Djukic et al., 2006; Ginhoux et al., 2013). We used a well established method to differentiate RPCs from hESCs (Lamba et al., 2006, 2010) and then treated the resultant RPCs with Notch ligands to drive them to differentiate into glial cells. As there is no sole Müller cell marker available and GFAP is expressed by both Müller cells and astrocytes in the neural retina, we are unable to identify the exact subtype of retinal glial cells we have differentiated in this study. However, since the GFAP^+^ cells were derived from RPCs, we believe most GFAP^+^ cells that we produced represent a Müller cell population rather than astrocytes. Future studies to identify Müller cell-specific markers and conduct Müller cell functional assays, such as measurement of aquaporin 4-mediated osmotic water permeability and electrophysiological recording of Kir4.1 current-to-voltage relationship (Reichenbach and Bringmann, 2013), will help determine the composition and function of differentiated glial cells resulting from Notch ligand treatment.

Among the markers we used to characterize glial cell differentiation, GFAP, a pan glial cell marker, showed the most profound upregulation over the course of promoting the Notch signaling pathway. We also noticed that the level of other glial cell markers, including GS, CRALBP and vimentin, remained relatively low in RPCs after Notch ligand treatment. Perhaps RPCs require a longer-term of manipulation of the Notch signaling pathway to express the full panel of Müller cell markers. Previous studies indicate that, among different populations of retinal cells during development, Müller cells are the last retinal cell type to be formed (Cepko, 1993; Vecino and Acera, 2015). Consistent with these findings, it has been reported that it takes approximately 4–5 months to form Müller cells during the differentiation of 3 dimensional retinal organoids from hESCs (Zhong et al., 2014).

While Notch treatment substantially increased GFAP gene expression in RPCs, we also observed a decrease in GFAP expression 6 weeks after Notch treatment compared with earlier treatment (Figure 2C). Interestingly, the Notch downstream effector genes including *Hes1* and *Hes5*, also decreased over time (Supplementary Figure S3). As mature Müller glia in the normal retina express little GFAP (Bringmann et al., 2006; Beach et al., 2017), the reduced GFAP expression over time suggest that RPCs-derived retinal glia may become more mature at 6 weeks than 2 weeks after Notch treatment.

We observed that GFAP^+^ cells showed different morphologies after RPCs were treated with Notch ligands for the same duration (Figures 2E,H). Previous studies reported that Müller cells isolated from human retinas can have diverse morphologies during culture, including long spindle shape with processes and trapezoidal shape (Lupien et al., 2004; Giannelli et al., 2011). It has been reported that Müller cells in the macula display different morphology and function from peripheral Müller cells (Yamada, 1969; Gass, 1999; Bringmann et al., 2006; Zhang et al., 2019). We also recently found that Müller cells isolated from the macula are small spindle to stellate shaped cells with lower cytoplasm/nucleus ratios and shorter processes, while the Müller cells from peripheral retinas have larger cell bodies, multiple cytoplasmic processes, higher cytoplasm/nucleus ratios (Zhang et al., 2019).

We observed that inhibiting Notch signaling in RPCs led to upregulation of retinal neuronal markers including CRX and OTX2, with glial cell markers hardly detectable. This is consistent with a previous observation that inhibiting Notch signaling in postnatal murine eyes suppresses glial cell maturation (Vecino et al., 2016). These results indicate that both humans and rodents may share similar mechanisms in differentiating RPCs into retinal glial cells.

There are a number of limitations in this study. We used one glial cell surface marker (GFAP) for FACS analyses to study the number and proportion of glial cells differentiated from RPCs. This can be improved by using a panel of glial cell surface markers such as CD44 and CD29 in future experiments (Shinoe et al., 2010; Eastlake et al., 2019). Our study indicates that the Notch signaling pathway plays a pivotal role in driving hESCs-derived RPCs to differentiate into glial cells. Further studies are warranted to validate our differentiation method in multiple stem cell lines including induced pluripotent stem cells (iPSCs).

In summary, we have developed a method to differentiate human ESC-derived RPCs into retinal glial cells by promoting the Notch signaling pathway. Application of this differentiation method to patient-derived iPSCs will help advance the generation of stem cell disease models to study the cellular and molecular mechanisms of retinal diseases associated with glial dysfunction such as MacTel2. Our method may also be useful for developing therapeutic strategies such as drug screening and gene editing using patient-derived retinal glial cells.

## Data Availability Statement

The datasets generated for this study are available on request to the corresponding author.

## Ethics Statement

Experiments were performed in accordance with the 2010 Amendments to the National Academies’ Guidelines for Human Embryonic Stem Cell Research. The protocol was approved by the Human Ethics Committee in The University of Sydney (Project# 2015/142).

## Author Contributions

SC and MG conceived the project. SC and BY conducted the experiments. SC, WS, and MG analyzed the results and wrote the manuscript. SC, WS, KD, AP, RW, and MG critically revised the manuscript.

## Conflict of Interest

The authors declare that the research was conducted in the absence of any commercial or financial relationships that could be construed as a potential conflict of interest.

## Supplementary Material

FIGURE S1 RT-PCR analyses of changes in expression of stem cell and retinal progenitor genes during RPC differentiation. **(A,B)** Stem cells markers were gradually decreased from day 0 to day 12, whereas increased expression of a panel of RPC markers were observed as early as at day 3 of RPC differentiation.

FIGURE S2 Bright field images showing cell morphologies over the course of RPC differentiation. **(A)** Morphology of RPCs differentiated from H9 ESCs during culture without Notch ligand treatment. **(B)** Morphology of RPCs treated with Notch ligands for 1–6 weeks.

FIGURE S3 qPCR analysis of Notch downstream effector genes 6 weeks after Notch stimulation in RPCs. We found decreased expression of Hes1 and Hes5 6 weeks after Notch promotion despite adding fresh Notch ligands every 2–3 days while changing media. Boxes represents the interquartile range, or the middle 50% of observations. The dotted line represents the median gene expression. Whiskers represent the minimum and maximum observations *n* = 3, *p* > 0.05.

FIGURE S4 FACS analyses of GFAP^+^ cells 4 and 6 weeks after Notch ligand treatment in H9-derived RPCs. The number of GFAP^+^ cell was counted from 10,000 events. **(A,C)** Notch treatment for 4 weeks increased the number of GFAP^+^ cells compared with un-treated RPCs cultured for 7 weeks. **(B,D)** There was a decrease in the number of GFAP^+^ cells in both groups after incubation in test media for further 2 weeks. Error bars represent SEM, *n* = 4/group.
