# Supplementary figures and images for: Differentiation of Retinal Glial Cells From Human Embryonic Stem Cells by Promoting the Notch Signaling Pathway
Source: Front Cell Neurosci. 2019 Dec 3;13:527. doi: 10.3389/fncel.2019.00527 (PMC6901827; doi:10.3389/fncel.2019.00527)

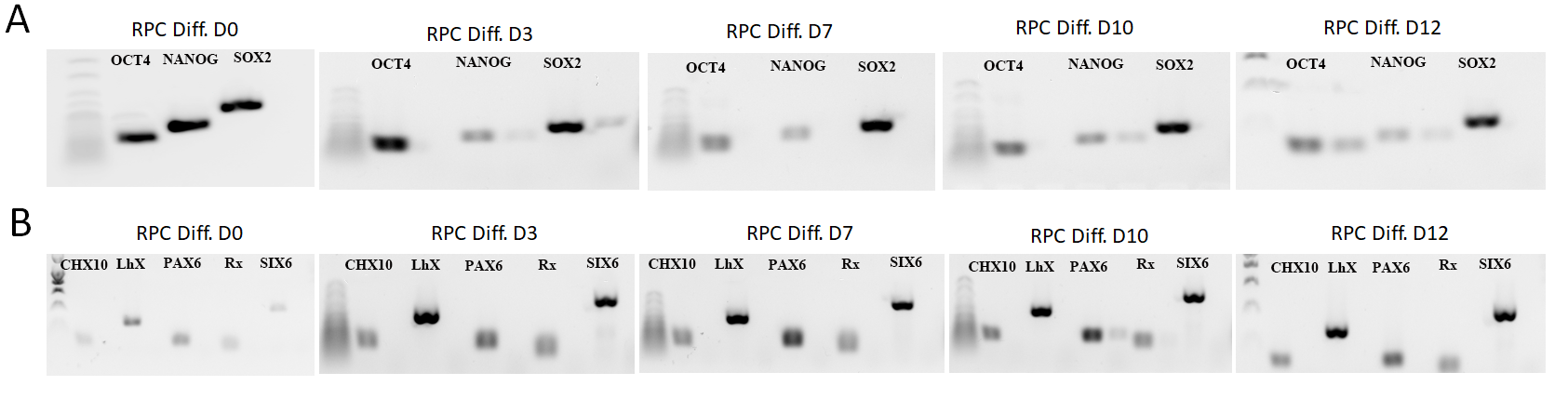

Supplement: FIGURE S1 — RT-PCR analyses of changes in expression of stem cell and retinal progenitor genes during RPC differentiation. (A,B) Stem cells markers were gradually decreased from day 0 to day 12, whereas increased expression of a panel of RPC markers were observed as early as at day 3 of RPC differentiation. [file Image_1.TIF]

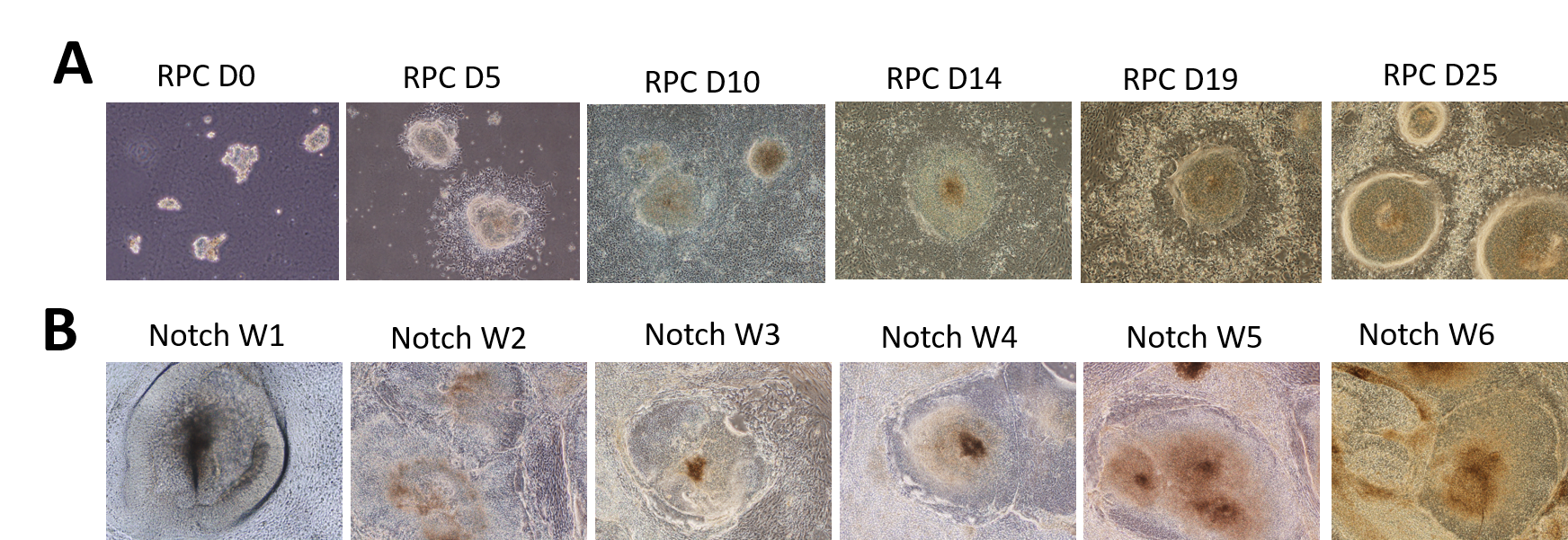

Supplement: FIGURE S2 — Bright field images showing cell morphologies over the course of RPC differentiation. (A) Morphology of RPCs differentiated from H9 ESCs during culture without Notch ligand treatment. (B) Morphology of RPCs treated with Notch ligands for 1–6 weeks. [file Image_2.TIF]

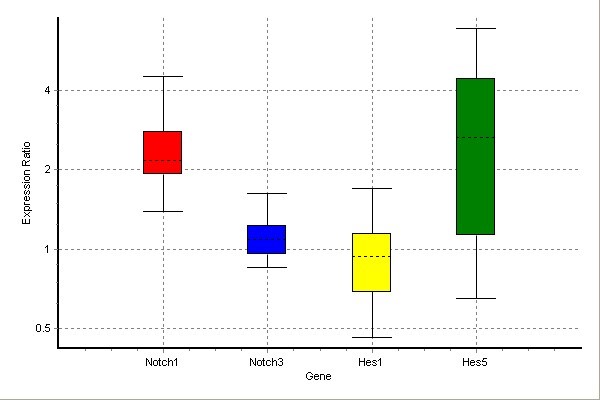

Supplement: FIGURE S3 — qPCR analysis of Notch downstream effector genes 6 weeks after Notch stimulation in RPCs. We found decreased expression of Hes1 and Hes5 6 weeks after Notch promotion despite adding fresh Notch ligands every 2–3 days while changing media. Boxes represents the interquartile range, or the middle 50% of observations. The dotted line represents the median gene expression. Whiskers represent the minimum and maximum observations n = 3, p > 0.05. [file Image_3.JPEG]

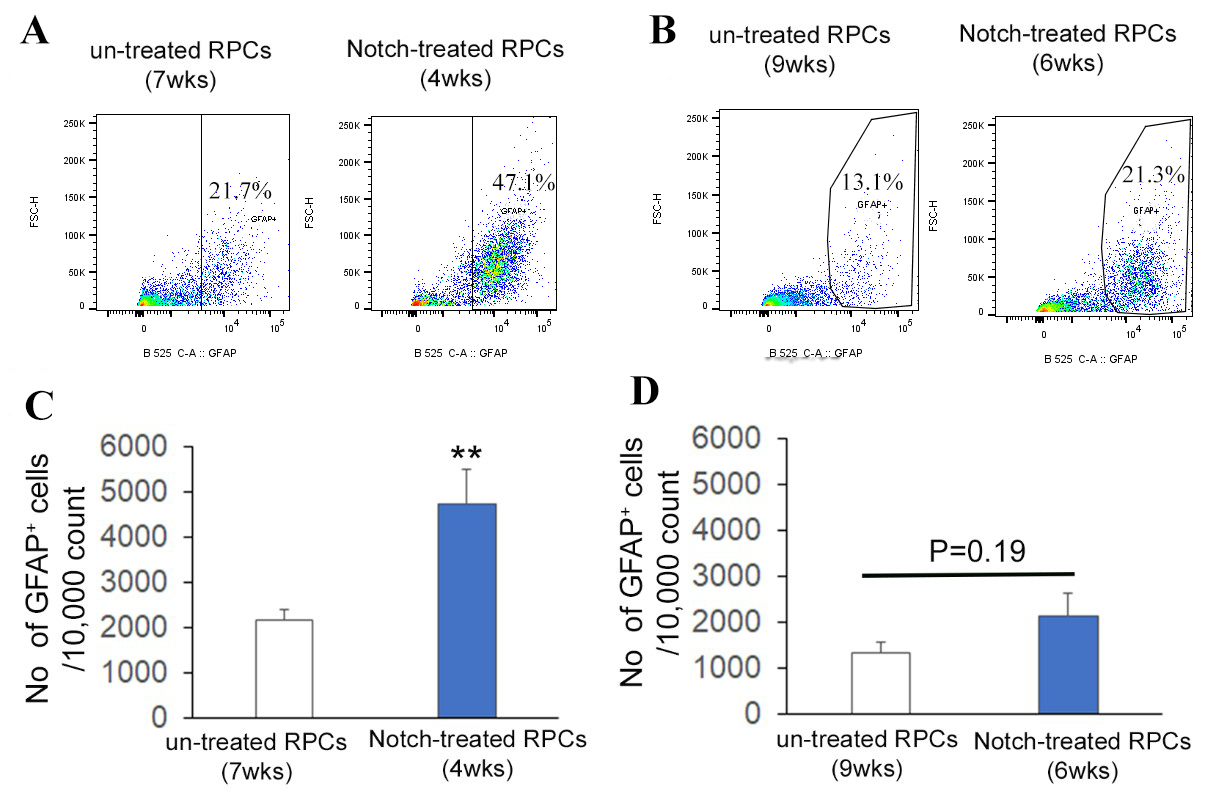

Supplement: FIGURE S4 — FACS analyses of GFAP+ cells 4 and 6 weeks after Notch ligand treatment in H9-derived RPCs. The number of GFAP+ cell was counted from 10,000 events. (A,C) Notch treatment for 4 weeks increased the number of GFAP+ cells compared with un-treated RPCs cultured for 7 weeks. (B,D) There was a decrease in the number of GFAP+ cells in both groups after incubation in test media for further 2 weeks. Error bars represent SEM, n = 4/group. [file Image_4.TIF]
